# Modeling seasonal immune dynamics of honey bee (*Apis mellifera* L.) response to injection of heat-killed Serratia marcescens

**DOI:** 10.1371/journal.pone.0311415

**Published:** 2024-10-04

**Authors:** Jana Hurychová, Jakub Dostál, Martin Kunc, Sara Šreibr, Silvie Dostálková, Marek Petřivalský, Pavel Hyršl, Dalibor Titěra, Jiří Danihlík, Pavel Dobeš

**Affiliations:** 1 Department of Experimental Biology, Faculty of Science, Masaryk University, Brno, Czech Republic; 2 Department of Mathematical Analysis and Application of Mathematics, Faculty of Science, Palacký University Olomouc, Olomouc, Czech Republic; 3 Department of Biochemistry, Faculty of Science, Palacký University Olomouc, Olomouc, Czech Republic; 4 Department of Zoology and Fisheries, Faculty of Agrobiology, Food and Natural Resources, Czech University of Life Science Prague, Prague, Czech Republic; University of Murcia: Universidad de Murcia, SPAIN

## Abstract

The honey bee, *Apis mellifera* L., is one of the main pollinators worldwide. In a temperate climate, seasonality affects the life span, behavior, physiology, and immunity of honey bees. In consequence, it impacts their interaction with pathogens and parasites. In this study, we used Bayesian statistics and modeling to examine the immune response dynamics of summer and winter honey bee workers after injection with the heat-killed bacteria *Serratia marcescens*, an opportunistic honey bee pathogen. We investigated the humoral and cellular immune response at the transcriptional and functional levels using qPCR of selected immune genes, antimicrobial activity assay, and flow cytometric analysis of hemocyte concentration. Our data demonstrate increased antimicrobial activity at transcriptional and functional levels in summer and winter workers after injection, with a stronger immune response in winter bees. On the other hand, an increase in hemocyte concentration was observed only in the summer bee population. Our results indicate that the summer population mounts a cellular response when challenged with heat-killed *S*. *marcescens*, while winter honey bees predominantly rely on humoral immune reactions. We created a model describing the honey bee immune response dynamics to bacteria-derived components by applying Bayesian statistics to our data. This model can be employed in further research and facilitate the investigating of the honey bee immune system and its response to pathogens.

## Introduction

In a temperate climate, two distinct populations of honey bee workers occur in colonies within the year cycle, i.e., short-living ‘summer’ and long-living ‘diutinus’ bees. These two populations differ in their life span and in fundamental physiological and immune parameters [1–3]. The seasonal changes in honey bee immune defenses are triggered by the varying spectrum of pathogens present in the honey bee environment.

Individual honey bee immunity is composed of a) anatomical barriers of the body, b) cell-based immunity, which is mediated by hemocytes and their interactions with invaders, and c) humoral immunity depending mainly on phenoloxidase, lysozyme, and antimicrobial peptides (AMPs) activity [4]. Since honey bees are social insects living in colonies, social immunity such as grooming, hygienic behavior, or social fever also plays an important role in protecting the colony and the individuals against pathogens and parasites [5].

The most threatening pathogens emanate from various taxa, including parasitic mites, fungi, bacteria, and viruses, often causing co-infections [6]. In the last decades, spreading epidemics of *Varroa destructor* compromised the health of honey bee colonies. The mite interferes with the development of bees, negatively influences bee immunity, and serves as a vector for viral pathogens [7–9]. Furthermore, it has been suggested that bacteria can also be transmitted into the honey bee hemolymph through the feeding wound [10–12].

The bacterial pathogens of honey bees are spread worldwide and can cause septicemia in both larvae and adult bees [13, 14]. Most of the attention has been focused on *Paenibacillus larvae* and *Melissococcus plutonius*, the causative agents of the American and the European foulbrood, respectively. *P*. *larvae* and *M*. *plutonius* are highly contagious bacteria, causing decimating colony losses of managed bees. Other recognized opportunistic pathogens of honey bees, *Spiroplasma apis*, *Spiroplasma melliferum*, *Hafnia alvei*, *Klebsiella* spp., *Enterobacter* spp., and *Serratia marcescens*, are yet to be studied [15, 16]. Under normal conditions, these opportunistic bacteria coexist with their host in a commensal relationship; however, they can become pathogenic when the host’s immune system is compromised by factors such as pesticide exposure [17, 18], antibiotic treatment [19] or the presence of other pathogens that reduce immune defense [20].

In previous research, viable *S*. *marcescens* was detected in Varroa mites sampled from hives infected with this bacterium [13]. Therefore, Varroa mites were suggested as a possible vector for transferring *S*. *marcescens* into the bee hemolymph, causing sepsis and death of honey bee workers [13]. Moreover, some strains of this opportunistic pathogen, normally present in low abundance in the honey bee gut, may perturb the epithelial barrier, enter the hemocoel, and become highly virulent [20, 21]. This virulence is exacerbated by co-infection with *Nosema ceranae*, which disrupts gut epithelial cells [20].

Former studies discovered variable immune system reactions of larvae and adults at different life stages or castes after pathogen stimulation [9, 22–24]. Previously, differences between summer and winter (diutinus) bee populations in response to heat-killed bacteria were described [3]. The basal relative gene expression of antimicrobial peptides was higher in summer bees; however, the immune response of winter bees, including hemolymph antimicrobial activity, was more pronounced. These results were obtained by sampling bees at a single time point, providing a general view of the seasonal specificity of bee immune responses but without insights into dynamics within a prolonged period after immune activation stimuli.

In this study, we sampled bees at several time points to describe time-dependent changes in cellular and humoral immune parameters after injecting heat-killed bacteria *S*. *marcescens*. To effectively approximate the dynamics of expression or production of immune factors, a Bayesian approach was used. Bayesian statistics use available knowledge about a given parameter in a statistical model and update the model with provided observational data to determine the posterior distribution [25]. In this study, the model based on the scaled derivative of logistic functions enables the identification of important parameters in the dynamics of the measured variables, i.e. the strength (amplitude) of the analyzed immune factors and the time when the peak of the response occurs, which might be overlooked with original separated measurements. This approach provides a foundation for future investigations into the nuanced dynamics of honey bee immune responses to pathogens.

## Material and methods

### Experimental honey bees

The honey bee workers, *Apis mellifera* L., were collected from three colonies led by unrelated queens, kept at the apiary Kývalka, Czech Republic (49.1886747°N, 16.4513211°E). The colonies were maintained according to standard beekeeping practice by a professional beekeeper. Specifically, they were fed approximately 15 kg of sucrose solution (3:2 sucrose/water) for wintering, treated with flumethrin against *Varroa destructor*, and regularly inspected for varroosis or nosemosis. Clinical signs of varroosis and nosemosis (presence of *Nosema* spp. in the gut) were not observed in any of the experimental colonies. For the experiment, short-living summer bees were collected in July 2020, whereas long-living winter bees (i.e. diutinus) were collected at the end of September 2020. Honey bees taken in September from the same location and apiary were previously characterized as winter bees [2, 3]. The honey bees used in the study were age-synchronized to 10 days, according to Dostálková et al. [3]. Shortly, six frames (two from each hive) with capped brood were selected approximately one to three days before uncapping, placed in frame cages, and kept in the respective hives until the young bees emerged. The newly emerged bees were marked with color on their thoraces and returned to their colonies. After 10 days, marked bees were recollected (2,965 in summer and 1,870 in winter) and transported in a well-ventilated plywood box to the laboratory. Transport took approximately 20 minutes, and the bees were immediately processed in the laboratory as follows.

### Honey bees treatment

The bees were pooled and randomly divided into four groups. Bees in the Control group were left without any treatment, whereas the CO_2_ group was anesthetized with gaseous carbon dioxide. Bees in the PBS and Bacteria groups were also anesthetized with CO_2_ and subsequently injected using a Hamilton syringe (Hamilton, Reno, NV, USA) in the dorsal part of the abdomen with 5 μl of sterile phosphate buffered saline (PBS; pH 7.0; Sigma-Aldrich, St. Louis, MO, USA) or 5 μl of heat-killed *Serratia marcescens* CCM 303 in PBS (100 bacterial cells/μl), respectively. The *Serratia* strain CCM 303, from the Czech Collection of Microorganisms, was cultivated on standard LB agar (Roth, Karlsruhe, Germany) at room temperature. The newly grown colonies were then resuspended in PBS to the required density and inactivated in a water bath at 80°C for 30 minutes. The bee abdomens were surface-sterilized with 5 μl of 96% ethanol prior to injection. Bees from all four groups were housed in plastic cages (100 bees/cage/0.5 dm^3^) prepared according to Kunc et al. [26] and kept at 34°C with sucrose solution (1:1 w/v; sucrose/water) provided *ad libitum*. Samples from summer bees were collected at 4, 8, 12, 16, 20, and 24 hours after the treatment. Due to the smaller number of emerging worker bees at the end of September, winter bees were sampled only at 4, 12, 20, and 24 hours post-treatment. Approximately one cage per treatment with 100 bees was sampled at each time point.

### Samples collection

Hemolymph samples were collected according to Kunc et al. [2]. Briefly, the abdomen was cut off, and 2 μl of hemolymph was taken from a drop appearing from a gently pressed thorax. The hemolymph was processed according to the specific analysis. For hemocyte quantification, the hemolymph from five bees was pooled, and processed immediately. For Apidaecin 1 quantification, hemolymph from individual bees was diluted 10× with 0.1% trifluoroacetic acid. For ELISA quantification of peptides, pooled hemolymph from five bees was also diluted 10× with 0.1% trifluoroacetic acid. For antimicrobial assay, hemolymph pooled from ten bees was diluted 1.25× with phenylthiourea (1 mg/ml in PBS) to prevent coagulation and melanization, and then stored at -80°C. The remaining bee abdomens were kept at -80°C for further RNA extraction and analysis of the relative gene expression of selected AMPs.

### Determination of antimicrobial activity

Antimicrobial activity in hemolymph treated with phenylthiourea was determined by radial diffusion on agar plates with *Micrococcus luteus* (CCM 169) as previously described [2]. Briefly, the bacterial suspension was cultured overnight in liquid LB medium (MOBIO, Carlsbad, CA, USA) with constant agitation (200 rpm; room temperature). The culture was diluted to OD_600_ value of 1.5 and mixed with melted LB agar (4% agar in LB; MOBIO, Carlsbad, CA, USA) in a ratio of 1:500 (v/v). Five hemolymph samples per treatment and sampling time point were used, and 5 μl of hemolymph applied to wells prepared in agar plates supplemented with *M*. *luteus*. The resulting inhibition zones around the wells were measured after 24 hours of incubation at 30°C. The antibacterial activity of hemolymph was quantified using lysozyme standards (EC 3.2.1.17; Sigma-Aldrich, St. Louis, MO, USA).

### Quantification of relative gene expression

Pools of five bee abdomens were homogenized for RNA extraction. The abdomens were smashed in plastic bags (Bioreba, Reinach, Switzerland) with a pestle in 1 ml of guanidinium isothiocyanate lysis buffer with 1% β-mercaptoethanol (Sigma-Aldrich, St. Louis, MO, USA) [27]. RNA isolation, cDNA synthesis, and qPCR were performed according to Dostálková et al. [3] with several modifications: reverse transcription was performed with an iScript^™^ cDNA Synthesis Kit (BioRad, Hercules, CA, USA) in 10 μl reaction volumes according to the manufacturer’s protocol. The expected PCR product size was tested by gel electrophoresis in 2.5% (w/v) agarose gel with detection by GelRed^®^ Nucleic Acid Gel Stain, 10000× (Biotium, Fremont, CA, USA) and with a 50–1000 bp PCR Marker (Promega, Madison, WI, USA). To ensure the RNA samples were free of DNA contamination, we used RNA isolates as templates for PCR with the housekeeping gene EF-1 alpha. The PCR results were then visualized using agarose gel electrophoresis. For all RNA samples there was no PCR product which indicated genomic DNA contamination. The relative expression of genes of interest (*apidaecin type 14*, *abaecin*, *defensin 1* and *hymenoptaecin*) to reference genes (*RPS-5*, *EF-1 alpha*) was calculated according to Pfaffl [28]. Briefly, the Ct values were obtained for both the gene of interest and the reference genes, as well as the efficiency of the qPCR reaction for each gene. The individual gene expression was calculated by multiplying its efficiency by the Ct value. The relative gene expression was then determined by normalizing reference gene expression to the expression of the gene of interest. The final value represents the geometric mean of the ratios for the two reference genes. This allows for accurate relative quantification of the target gene expression levels, corrected for the reference genes’ expression as internal controls. The calculated relative gene expression is independent of the control group, allowing for multiple comparisons across different experimental groups. This normalization to reference genes ensures that variations in gene expression can be compared across conditions without relying on a specific control group, enabling robust and consistent analysis. See S1 Table for primer sequences, efficiencies, and the formula used for the calculation of the relative gene expression. Nine to ten samples were analyzed per each treatment and sampling time point.

### Quantification of Apidaecin 1 in the hemolymph by LC-MS analysis

Apidaecin 1 was quantified in the 2 μl hemolymph of individual bees, diluted 10× with 0.1% trifluoracetic acid. Seven to sixteen samples were prepared for each condition. Before measurement, the hemolymph samples were lyophilized and subsequently dissolved in 20 μl 5% formic acid prior to UHPLC-MS analysis. Apidaecin 1 was quantified on a UHPLC-QTOF system Dionex Ultimate 3000 UHPLC system (Thermo Fisher Scientific, Waltham, MA, USA) coupled with a Compact qTOF mass spectrometer (Bruker Daltonics, Bremen, Germany) with electrospray ionization. Apidaecin 1 was quantified using an isotopically [^13^C_6_^15^N_4_] labeled internal standard of Apidaecin 1A (purity >98%; Clonestar Peptide Services, Brno, Czech Republic) according to Danihlík et al. [29]. The LC-MS method set-up was according to Dostálková et al. [3].

### Quantification of Abaecin, Defensin 1, and Hymenoptaecin by ELISA

Hemolymph pooled from five bees, diluted 10× with 0.1% trifluoracetic acid was used for the ELISA assay performed on a Corning^®^ 96 Well EIA/RIA Assay Microplate (Corning Inc., Somerville, MA, USA). Three samples per treatment and sampling time point were prepared. Before measurement, the hemolymph samples were lyophilized and subsequently dissolved in 600 μl of coating buffer. The volume of 100 μl of diluted samples, blanks (coating buffer), positive controls (peptide epitope supplied by the antibody manufacturer), negative controls (hemolymph from freshly emerged bee), and calibrators were pipetted per ELISA well and incubated at 4°C overnight for antigen binding. The next day, the ELISA plates were washed three times with 1× washing buffer with 0.1% (v/v) Tween 20. The unspecific binding was blocked with 200 μl of 0.5% non-fat milk (Sigma-Alrich, St. Louis, MO, USA) and incubated for 2 hours at 37°C, then again three times washed with washing buffer with 0.1% (v/v) Tween 20. The primary polyclonal rabbit antibodies (Clonestar Peptide Services, Brno, Czech Republic) were diluted in 1× washing buffer as follows: 1:500 for Defensin 1, 1:250 for Abaecin, and 1:500 for Hymenoptaecin antibody. Subsequently, the plate was incubated at 37°C for one hour, washed three times with washing buffer, and afterwards incubated at 37°C for one hour with secondary antibody (goat anti-rabbit IgG (whole molecule)-peroxidase conjugate, Sigma-Aldrich, St. Louis, MO, USA) diluted 1:3000 in 1× washing buffer with 0.1% (v/v) Tween 20. Then the plate was washed again four times with 1× washing buffer with 0.1% (v/v) Tween 20 and incubated in the dark at 37°C for one hour with 100 μl of the 2.4 mM tetramethylbenzidine substrate in phospho-citrate buffer with sodium perborate. The reaction was stopped by adding 50 μl of 0.5M H_2_SO_4_ to each well. The absorbance was recorded at 450 nm in a Synergy HT microplate reader (BioTek, Winooski, VT, USA). See S1 File for details on the preparation of ELISA buffers and reagents.

### Hemocytes quantification

The concentration of circulating hemocytes was analyzed according to Kunc et al. and Marringa et al. [26, 30]. Shortly, 10 μl of fresh hemolymph pooled from five bees were 50× diluted in cold insect physiological saline (10 mM EDTA, 30 mM sodium citrate; pH 7.0), and stained with wheat germ agglutinin conjugated with fluorescein isothiocyanate (WGA-FITC; 1 μg/ml; Sigma-Aldrich, St. Louis, MO, USA) and propidium iodide (0.1 mg/ml; Sigma-Aldrich, St. Louis, MO, USA). The samples were incubated at room temperature for 15 min in the dark and measured using a spectral flow cytometer Northern Lights 3000 (Cytek Biosciences, Fremont, CA, USA). The results were processed in SpectroFlo^®^ software. WGA-FITC positive and propidium iodide negative events were gated within singlets and quantified as live hemocytes with intact membranes. Five to eight samples were prepared for each condition, i.e., each treatment and sampling time point.

### Statistics

Original data gained from beforementioned analyses were employed to create probability model using Bayesian statistics. A simple Bayesian model of the response was developed in pymc python package [31]. The responses are modeled as a scaled derivative of the logistic function:

$$f\left(x\right)=\frac{\text{1}}{1+exp\left(-x\right)}$$

$$y\left(x;ampl,\;loc,\;scale\right)=4∙ampl∙f\left(\frac{x-loc}{scale}\right)∙\left(1-f\left(\frac{\ldots }{\ldots }\right)\right)$$

The code is in S2 File.

**Fig 1:**
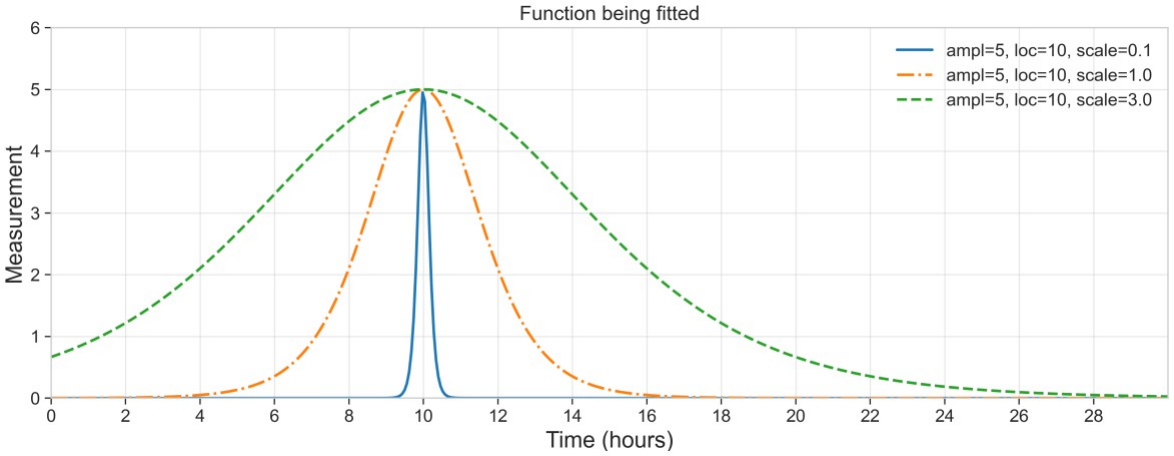
A selection of functions that are fitted to data. The figure shows the interpretation of the ‘ampl’ parameter, which corresponds to the highest point on the chart, and ‘loc’ parameter which corresponds to the time of maximal response. The ‘scale’ parameter is varied to show its effect on the speed of the response increase and decline.

This enables us to have three easily interpretable parameters (Fig 1):

- ampl: the highest value of the response, corresponds to the highest point on the chart;
- loc: the time point of the maximal response;
- scale: the speed of the response increase and decline (the model is symmetrical around the loc value).

**Fig 2:**
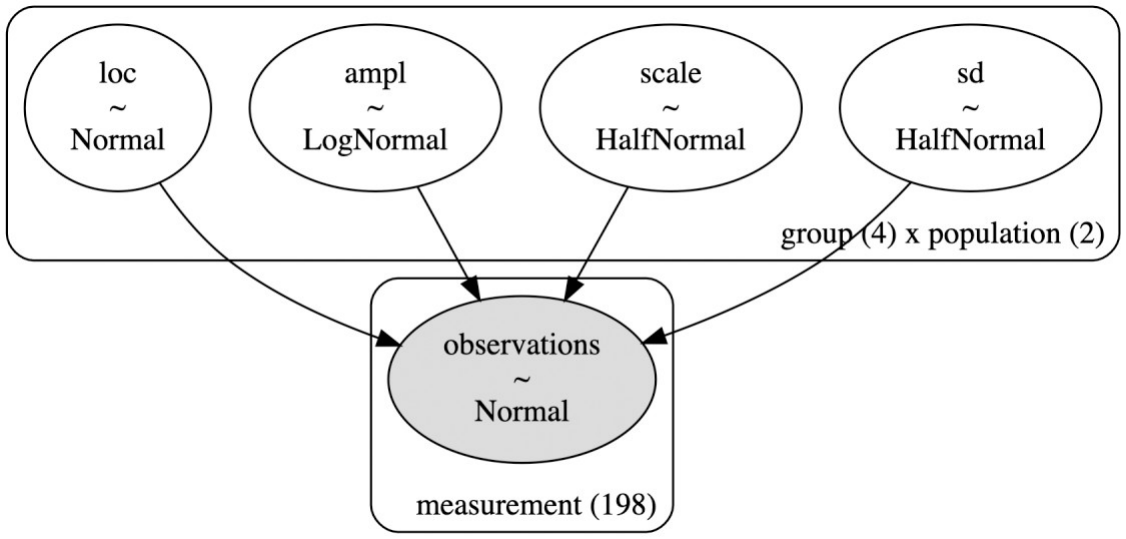
Illustration of the developed hierarchical Bayesian model. The parameters loc, ampl, scale, and sd, represents different prior distributions across the dimensions of four groups (Control, CO_2_, PBS, Bacteria) and two populations (Summer, Winter). The observed data is modeled using a normal distribution function of time and the aforementioned parameters, tailored to each subgroup within the data.

In Bayesian statistics, a prior is a probability distribution that represents the beliefs or information about a parameter before considering the current data. It expresses the uncertainty or prior knowledge about the parameter of interest. The prior distribution is updated with observed data to obtain the posterior distribution, which combines the prior information with the likelihood of the observed data to provide an updated estimate of the parameter. Our model is then specified using log-normal prior for the ampl parameter, gaussian prior for the loc parameter, and half-normal prior for the scale parameter (Fig 2). Each group and population have its own approximated parameters. The state-of-the-art No-U-Turn sampler [32] is then used to sample the posterior distribution of the parameters. The results are visualized in graphs showing ampl and loc at 95% Highest Density Interval (HDI). The values in the Result section describe the ampl or loc with 2.5–97.5% dispersion, i.e. 95% HDI, in the brackets. The original data are in S3 File.

## Results

### Antimicrobial activity

**Fig 3:**
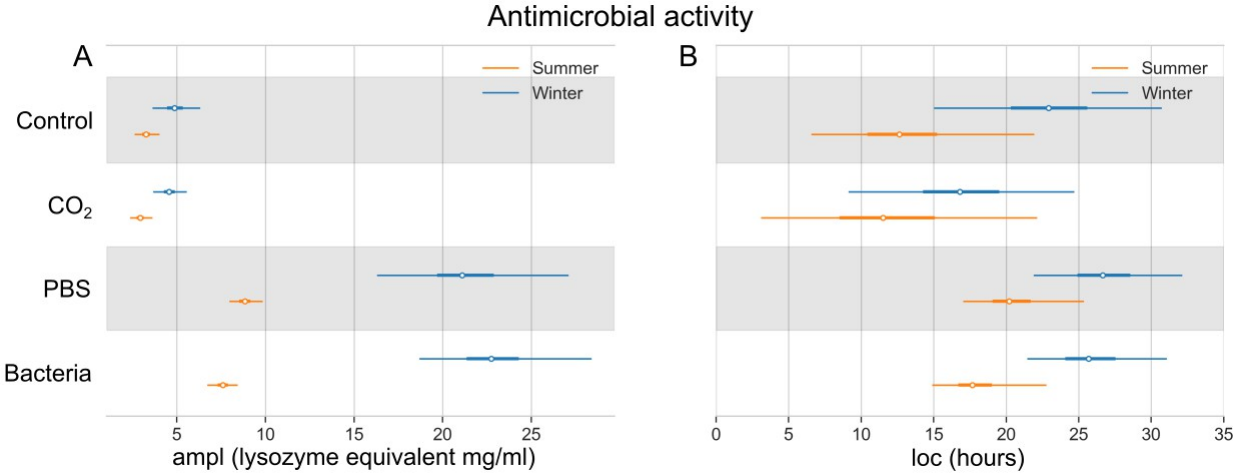
Bayesian model of antimicrobial activity measured as lysozyme equivalent (mg/ml). Comparison of four experimental groups, as well as summer (orange) and winter (blue) bee populations. (A) The highest values of the response (ampl; white dot) with 95% highest density interval (HDI; thin lines). (B) Time in hours of the maximal response (loc; white dot) with the 95% HDI (thin lines). The thick lines show 50% HDI.

The results obtained after the Bayesian modeling show a higher antimicrobial activity of honey bees in the winter compared to the summer population in all experimental groups (Fig 3A). The Control group shows a maximum of 4.94 (3.73–6.44) mg/ml of lysozyme in winter and 3.29 (2.62–4.04) mg/ml of lysozyme in the summer bee population (S2 Table). After the injection of heat-killed bacteria or PBS, the antimicrobial activity increased in both summer and winter bee populations compared to Control and CO_2_ group. Its maximal value in the Bacteria group is higher in winter bees, with a maximum of 22.94 (19.03–28.72) mg/ml of lysozyme in comparison to summer bees, with a maximum of 7.60 (6.74–8.46) mg/ml of lysozyme. The model predicts that winter bees need more time to respond and reach the peak of their antimicrobial activity (Fig 3B). The maximal value of antimicrobial activity after bacterial stimuli was reached at 18.0 (15.1–22.9) hours in summer and 25.9 (21.8–32.0) hours in winter bees. For probability curves of the immune response, please see S1–S6 Figs.

### Relative gene expression and concentration of selected antimicrobial peptides

**Fig 4:**
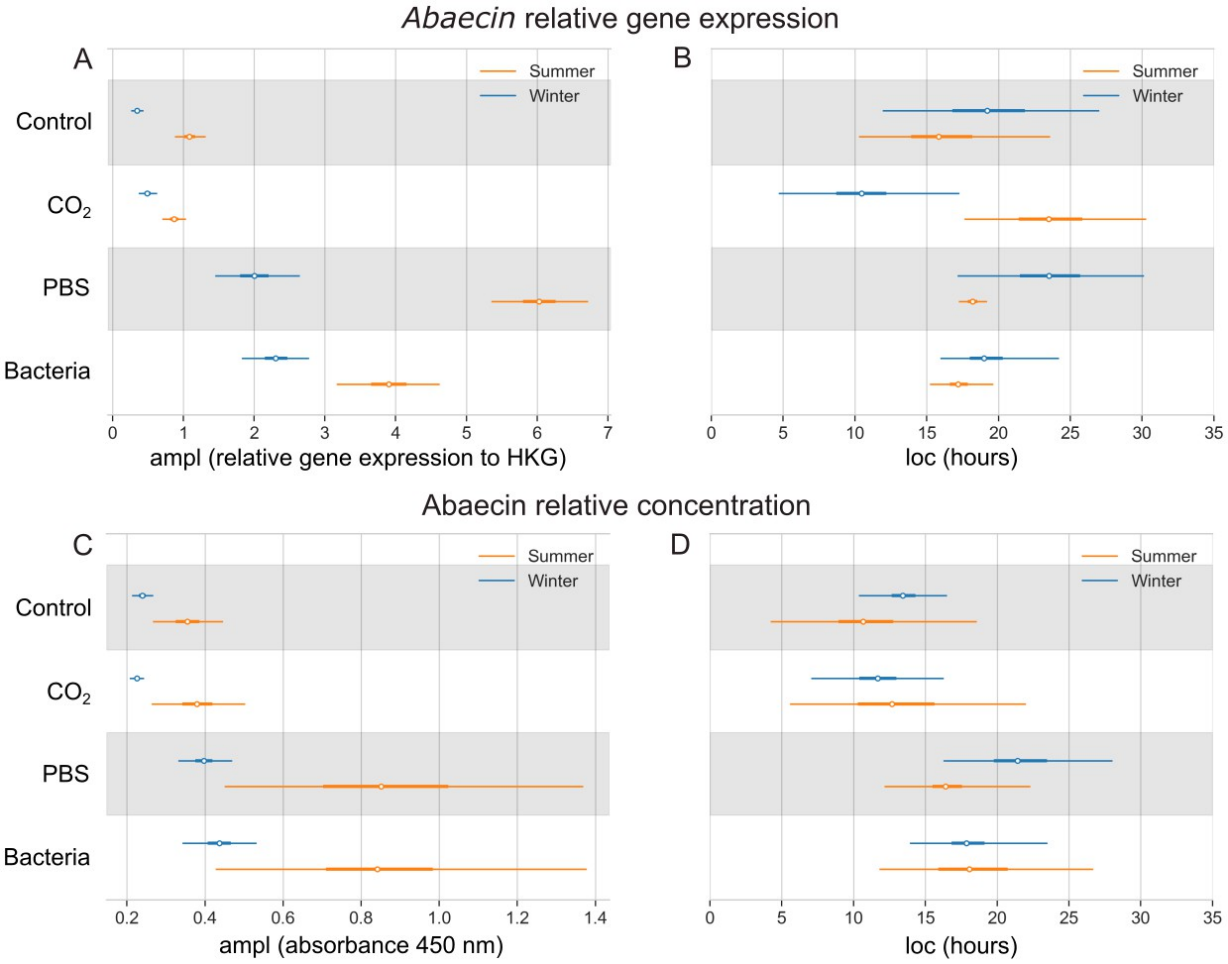
Bayesian model of *abaecin* relative gene expression and its relative peptide concentration in the hemolymph. Comparison of four experimental groups, as well as summer (orange) and winter (blue) bee populations. (A) The highest values of the *abaecin* relative gene expression (ampl; white dot) with the 95% highest density interval (HDI; thin lines). (B) Time in hours of the *abaecin* relative gene expression maximal response (loc; white dot) with the 95% HDI (thin lines). (C) The highest values of the Abaecin relative peptide concentration measured as absorbance at 450 nm (ampl; white dot) with the 95% HDI (thin lines). (D) Time in hours of the Abaecin relative peptide concentration maximal response (loc; white dot) with the 95% HDI (thin lines). The thick lines show 50% HDI.

In the Control group, *abaecin* relative gene expression is higher in summer, with maximal value of 1.09 (0.88–1.34), compared to 0.34 (0.25–0.45) in winter (Fig 4A). Following the injection of heat-killed bacteria, the values increased sharply, reaching 3.91 (3.16–4.70) in summer and 2.30 (1.84–2.81) in winter. The highest value of Abaecin relative concentration in the honey bee hemolymph is 0.36 (0.27–0.45) and 0.24 (0.21–0.26) in the Control summer and winter honey bee, respectively (Fig 4C). After the bacterial challenge, the values reach 0.85 (0.44–1.31) in summer and 0.44 (0.35–0.54) in winter. According to the Bayesian model, the maximal value of *abaecin* relative gene expression after bacterial stimuli is reached at 17.3 (15.5–20.0) hours in summer and 19.4 (16.1–25.1) hours in winter bees (Fig 4B). The relative concentration of Abaecin in the Bacteria group reaches its maximum at 18.4 (11.8–26.7) hours in summer and 18.1 (14.5–23.5) hours in winter honey bees (Fig 4D).

**Fig 5:**
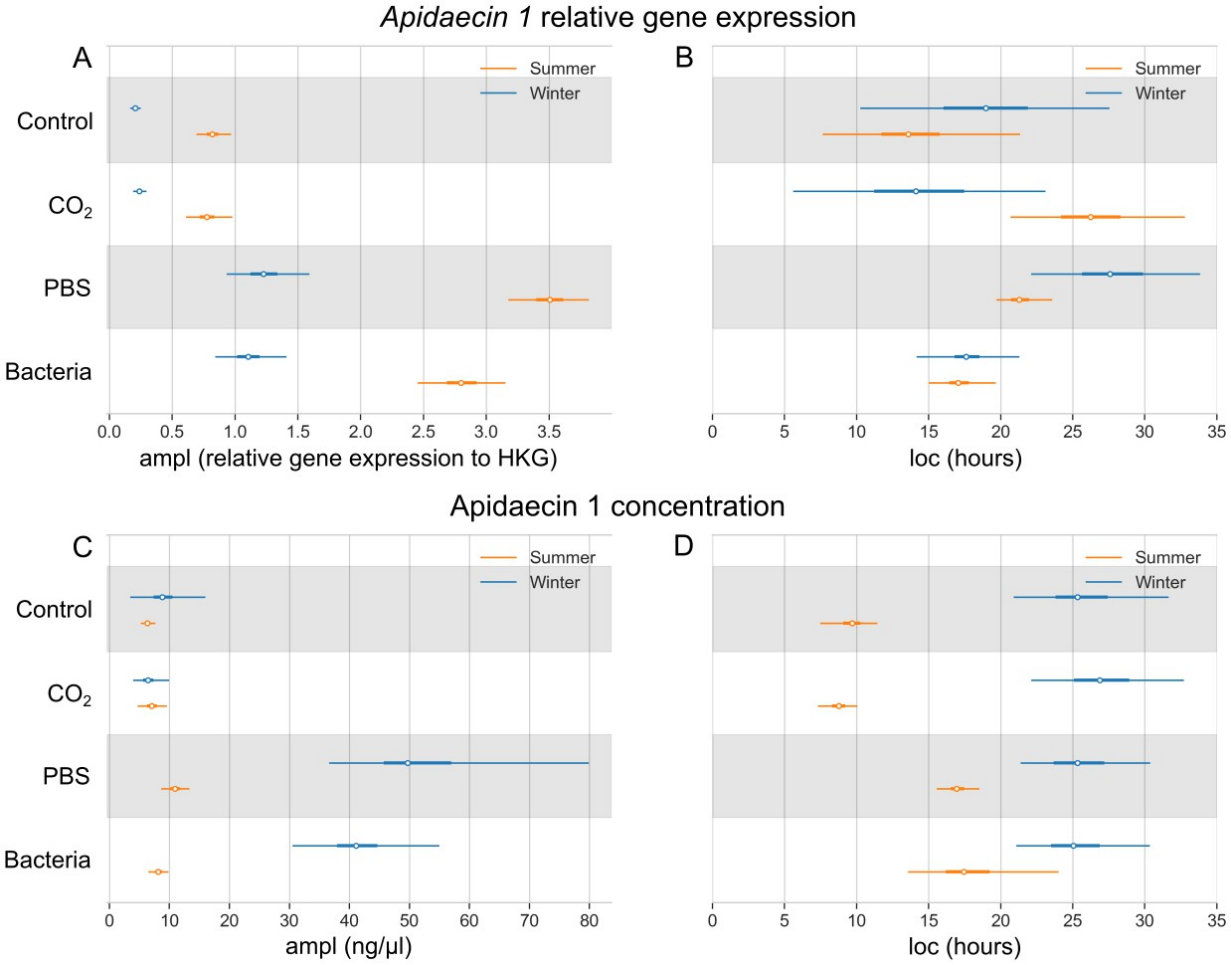
Bayesian model of *apidaecin 1* relative gene expression and its peptide concentration in the hemolymph. Comparison of four experimental groups, as well as summer (orange) and winter (blue) bee populations. (A) The highest values of the *apidaecin 1* relative gene expression (ampl; white dot) with the 95% highest density interval (HDI; thin lines). (B) Time in hours of the *apidaecin 1* relative gene expression maximal response (loc; white dot) with the 95% HDI (thin lines). (C) The highest values of the Apidaecin 1 peptide concentration in ng/μl (ampl; white dot) with the 95% HDI (thin lines). (D) Time in hours of the Apidaecin 1 peptide concentration maximal response (loc; white dot) with the 95% HDI (thin lines). The thick lines show 50% HDI.

The relative expression of *apidaecin 1* in the Control group reaches the highest value of 0.82 (0.69–0.97) in summer and 0.21 (0.17–0.25) in the winter (Fig 5A). Post the injection of heat-killed bacteria, the gene expression surged to 2.80 (2.46–3.16) in summer and 1.11 (0.85–1.42) in winter bees. Peptide concentration of Apidaecin 1 in the Control group is slightly lower in summer bees in comparison to winter bees, with a maximal value of 6.35 (5.05–7.68) ng/μl and 9.53 (4.68–18.92) ng/μl, respectively (Fig 5C). Its highest value in summer bees injected with bacteria is 8.21 (6.63–10.09) ng/μl, while in winter bees it reaches 41.97 (31.73–57.64) ng/μl. The highest value of the *apidaecin 1* relative gene expression after bacterial stimuli is reached at 17.2 (15.3–20.2) hours in summer bees and 17.8 (15.0–22.0) hours in winter bees (Fig 5B). The highest value of Apidaecin 1 concentration in honey bee hemolymph in the Bacteria group is reached at 18.0 (14.0–24.7) hours and 25.3 (21.4–31.7) hours in summer and winter bees, respectively (Fig 5D).

**Fig 6:**
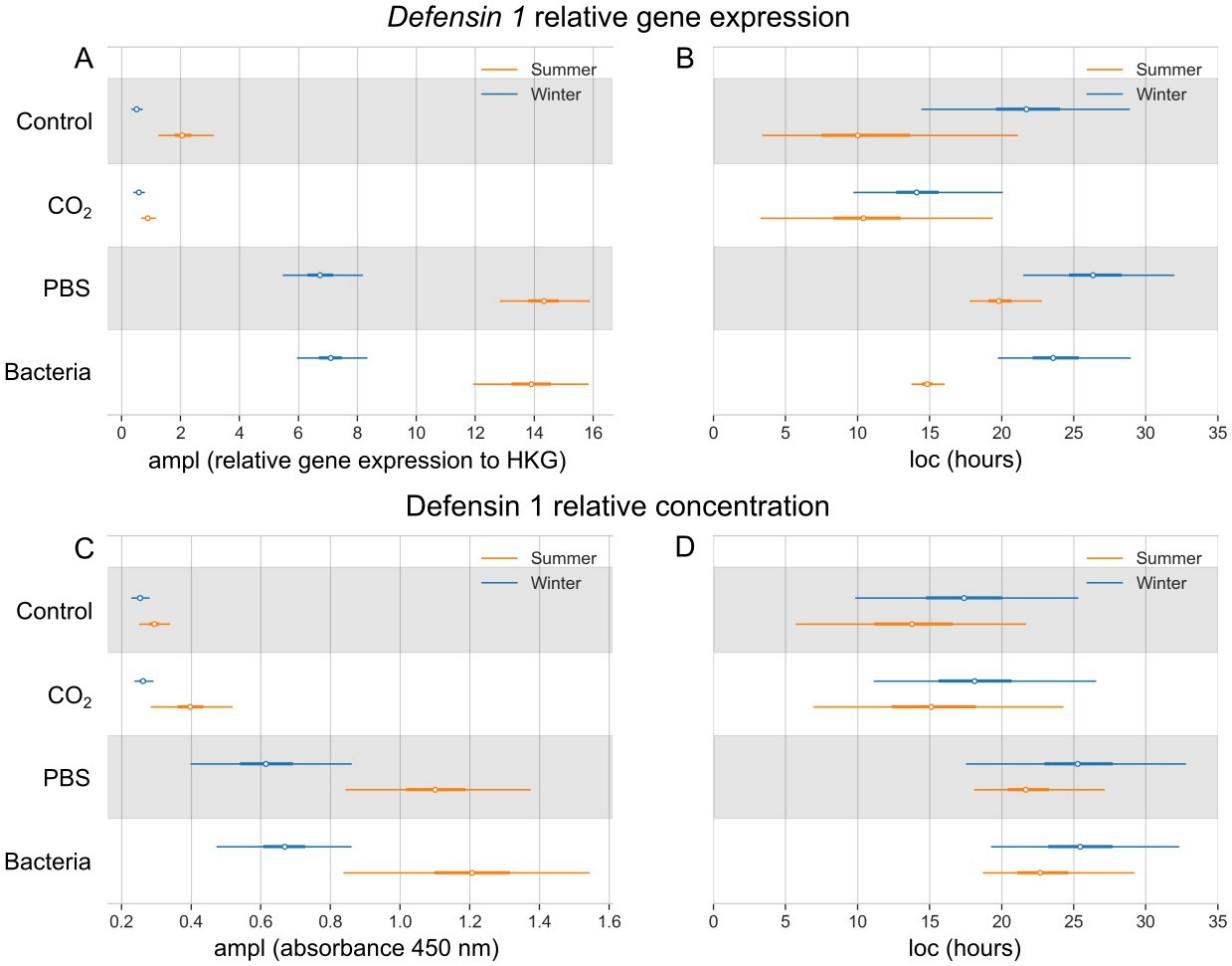
Bayesian model of *defensin 1* relative gene expression and its relative peptide concentration in the hemolymph. Comparison of four experimental groups, as well as summer (orange) and winter (blue) bee populations. (A) The highest values of the *defensin 1* relative gene expression measured as absorbance at 450 nm (ampl; white dot) with the 95% highest density interval (HDI; thin lines). (B) Time in hours of the *defensin 1* relative gene expression maximal response (loc; white dot) with the 95% HDI (thin lines). (C) The highest values of the Defensin 1 relative peptide concentration (ampl; white dot) with the 95% HDI (thin lines). (D) Time in hours of the Defensin 1 relative peptide concentration maximal response (loc; white dot) with the 95% HDI (thin lines). The thick lines show 50% HDI.

Relative gene expression of *defensin 1* in the Control group reaches the maximal value of 2.17 (1.36–3.82) in summer and 0.52 (0.35–0.73) in winter (Fig 6A). After bacterial challenge, the values rise to 13.90 (11.91–15.89) in summer and 7.13 (5.99–8.44) in winter. In the Control group, the highest value of Defensin 1 relative concentration in the honey bee hemolymph is 0.30 (0.25–0.34) in summer and 0.25 (0.23–0.28) in winter (Fig 6C). After the bacterial challenge the values reach 1.20 (0.81–1.57) in summer and 0.68 (0.50–0.87) in winter. According to the Bayesian model, the maximal value of *defensin 1* relative gene expression after bacterial stimuli is reached at 14.9 (13.7–16.0) hours in summer and 24.0 (20.2–29.5) hours in winter bees (Fig 6B). The relative concentration of Defensin 1 in the Bacteria group reaches its maximum at 22.9 (18.5–29.4) hours in summer and 25.8 (19.9–32.6) hours in winter bees (Fig 6D).

**Fig 7:**
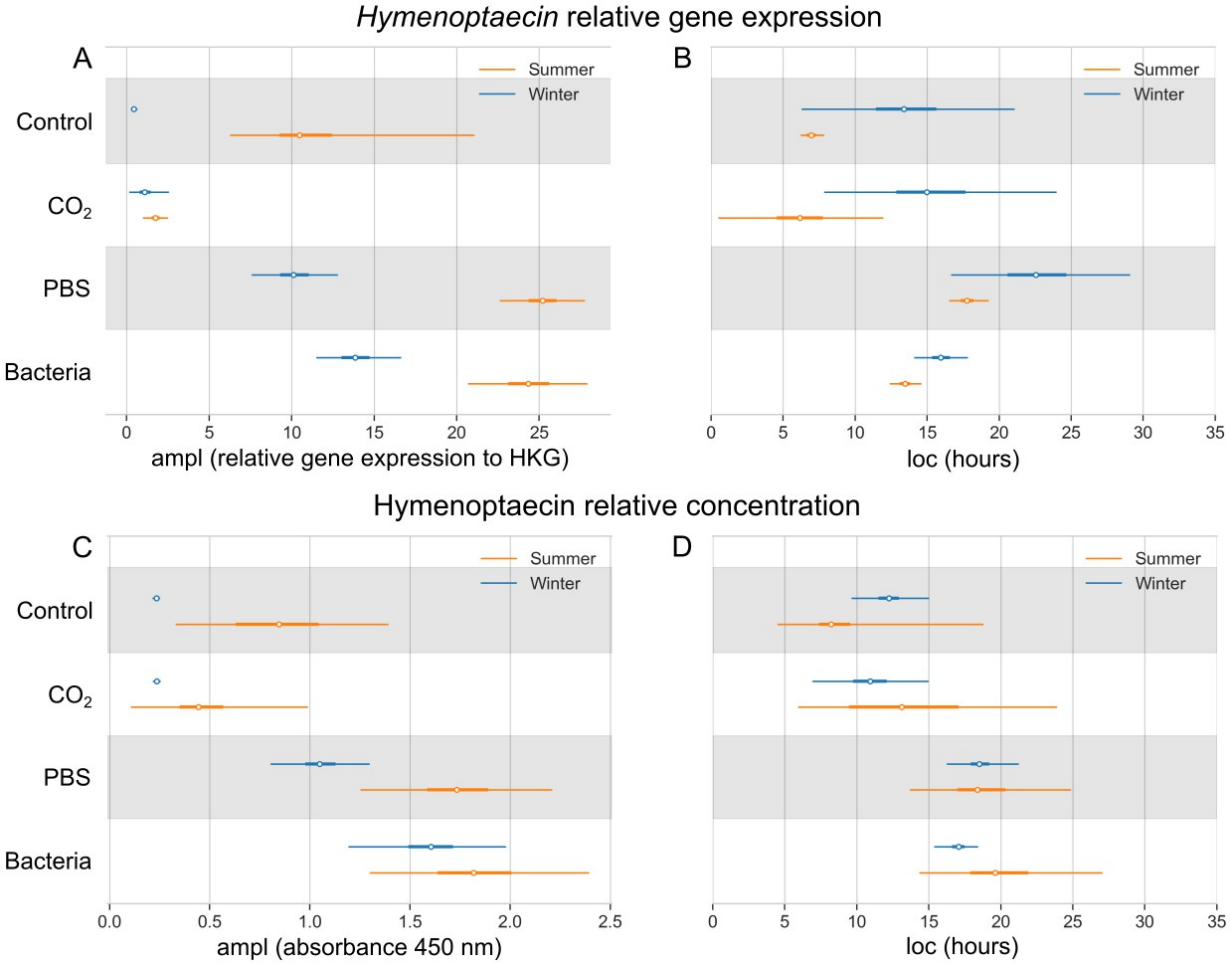
Bayesian model of *hymenoptaecin* relative gene expression and its relative peptide concentration in the hemolymph. Comparison of four experimental groups, as well as summer (orange) and winter (blue) bee populations. (A) The highest values of the *hymenoptaecin* relative gene expression (ampl; white dot) with the 95% highest density interval (HDI; thin lines). (B) Time in hours of the *hymenoptaecin* relative gene expression maximal response (loc; white dot) with the 95% HDI (thin lines). (C) The highest values of the Hymenoptaecin relative peptide concentration measured as absorbance at 450 nm (ampl; white dot) with the 95% HDI (thin lines). (D) Time in hours of the Hymenoptaecin relative peptide concentration maximal response (loc; white dot) with the 95% HDI (thin lines). The thick lines show 50% HDI.

*Hymenoptaecin* relative gene expression in the Control group reaches the maximal value of 12.05 (7.43–27.76) in summer and 0.44 (0.25–0.68) in winter (Fig 7A). After the bacterial challenge the values rise to 24.33 (20.83–27.83) in summer and 13.84 (11.32–16.53) in winter. The highest value of Hymenoptaecin relative concentration in the honey bee hemolymph is 0.84 (0.29–1.42) and 0.23 (0.22–0.25) in the Control summer and winter honey bee, respectively (Fig 7C). After the bacterial challenge, the values reach 1.82 (1.30–2.36) in summer and 1.60 (1.21–1.97) in winter. According to the Bayesian model, the maximal value of *hymenoptaecin* relative gene expression after bacterial stimuli is reached at 13.5 (12.4–14.6) hours in summer and 15.9 (14.0–17.8) hours in winter bees (Fig 7B). The relative concentration of Hymenoptaecin in the hemolymph of the Bacteria group reaches its maximum at 20.1 (14.6–27.1) hours in summer and 17.0 (15.4–18.4) hours in winter honey bees (Fig 7D). For probability curves of the immune response, please see S1–S6 Figs.

### Concentration of circulating hemocytes

**Fig 8:**
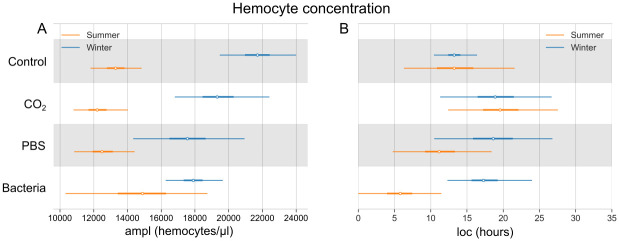
Bayesian model of hemocyte concentration (circulating hemocytes/μl of hemolymph) measured by flow cytometry. Comparison of four experimental groups, as well as summer (orange) and winter (blue) bee populations. (A) The highest values of the response (ampl; white dot) with the 95% highest density interval (HDI; thin lines). (B) Time in hours of the maximal response (loc; white dot) with the 95% HDI (thin lines). The thick lines show 50% HDI.

The Bayesian model shows that the concentration of hemocytes is overall higher in winter compared to summer bees (Fig 8A). In the Control group, the concentration reaches the highest value of 21730 (19380–24020) hemocytes/μl in the winter and 13300 (11900–14830) hemocytes/μl in the summer population. After injection of heat-killed bacteria we observed increased hemocyte concentration in summer bees (S6 Fig), with a maximum of 14790 (10590–19100) hemocytes/μl followed by a steady decrease. The highest value of hemocyte concentration in the summer population is reached at 5.9 (0.3–11.8) hours after injection with heat-killed bacteria (Fig 8B). For probability curves of the immune response, please see S1–S6 Figs.

## Discussion

Applying Bayesian statistics to describe the honey bee immune response dynamics to a heat-killed bacterial pathogen offers several advantages over relying on separated measurements, which often provide limited insights into infection dynamics. In an experiment where measurements are taken at specific time intervals, Bayesian statistics allow us to model the system behavior between these points, enabling us to estimate peak values that were not directly measured. Bayesian models integrate prior knowledge with current data, allowing for a more comprehensive analysis of the immune response. Bayesian methods also account for uncertainty and variability in the data, providing a probabilistic framework that quantifies the confidence in the findings. To obtain original data, we injected 10-day-old honey bees collected from the summer and winter populations with heat-killed bacteria *S*. *marcescens* and sampled their tissues for laboratory analyses at several time points after bacteria injection. Here, we present a model based on our experimental data that provides improved approximation of the dynamics of honey bee immune response to the heat-killed bacterial pathogen, compared to a commonly used frequentist statistical data evaluation.

The honey bee immune system consists of cellular and humoral immunity. The humoral response to bacteria includes mainly melanization, secretion of AMPs and other molecules with bactericidal or bacteriostatic functions. The increase in the honey bee antimicrobial activity can be triggered not only by live pathogens, but also by the injection of pathogen-associated molecular patterns such as lipopolysaccharide [22], the outer membrane component of gram-negative bacteria, or wounding by aseptic injection of saline buffer [24]. Here, we show an increase in antimicrobial activity in both summer and winter bees after the injection of either heat-killed gram-negative bacteria *S*. *marcescens* or PBS with similar progression observed in both cases. In line with our previous studies comparing summer and winter bee populations, we have demonstrated that the winter bees are characterized by a higher constitutive level and a stronger inducible capacity of antimicrobial activity [2, 3].

Previously, Gätschenberger et al. [33] detected antimicrobial activity in one-day-old honey bee workers injected with live *Escherichia coli* six hours post-treatment, showing a peak at 24 hours. This level of antimicrobial activity remained stable till the end of the experiment at 72 hours post-injection. In our study on 10-day-old bees, the Bayesian model shows the expected peak of the antimicrobial activity at 18.0 hours after the injection of heat-killed bacteria in summer bees, whereas the peak in the winter bees is reached at 25.9 hours with a presumption of prolonged duration. We hypothesize that the delay in reaching the maximum in the winter population may be due to the approximately three times stronger response mounted in the winter bees.

The key element of insect humoral defense are AMPs. In the hemolymph of honey bees infected with viable *E*. *coli*, four types of AMPs were described: Apidaecins [34], Abaecin [35], Hymenoptaecin [36] and Defensin [37]. In this study, the Bayesian model confirmed our previous findings on higher gene expression of AMPs in summer bees [3]. The immune response was activated even after an aseptic injection of saline buffer, which is in agreement with previous results [24, 38, 39]. Zhao et al. [40] reported induced expression of antibacterial peptides in bees, that were in contact with Varroa mites. It is not clear if the bee immune system was activated due to the observed increase of the deformed wing virus load, possible bacterial transfer through the wound caused by the feeding mite [10, 11], or the wound itself.

Apidaecins are assumed to be the major AMPs in honey bee hemolymph challenged with bacteria, mainly due to the presence of repetitive units in the gene precursor structure, which allows rapid amplification [41]. Previously, they were reported to be the main actors against gram-negative bacteria [34]. However, in the same study, Apidaecin 1 showed no or low effect against *S*. *marcescens*. In this study, the measured concentration of Apidaecin 1 in the hemolymph of summer honey bees after injection of heat-killed *S*. *marcescens* was rather moderate, with a maximum of 8.21 ng/μl, while the winter honey bees reached a maximum of 41.97 ng/μl of Apidaecin 1. In our previous study, when measured 24 hours post-infection by heat-killed *E*. *coli* and *P*. *larvae*, the concentration of Apidaecin 1 was 23.7 ng/μl in summer and 20.3 ng/μl in the winter population [3]. It could be hypothesized, that the discrepancy between concentrations might have been caused by different bacteria used in the study. Moreover, a higher concentration of Apidaecin 1 in winter bees caused by heat-killed *S*. *marcescens* in comparison to summer bees can indicate possible higher sensitivity to that pathogen during winter [21].

Casteels-Josson et al. [37] reported that transcripts of *defensin 1* occur in the honey bee hemolymph challenged with bacteria later (12 hours) than the transcripts of other AMPs and that the upregulation of *defensin 1* was minimal. On the other hand, Lourenço et al. [42] detected a statistically significant upregulation of *defensin 1* in bees injected with *S*. *marcescens* compared to non-injected bees already 5 hours post-treatment. In this study, a slow increase of *defensin 1* gene expression in winter honey bees with a peak at 24.0 hours after bacterial injection was observed. This was not the case in summer bees, which reached the maximal value at 14.9 hours under the same conditions. Although Defensin 1 was reported to act primarily against gram-positive bacteria [37], its gene expression was stronger compared to *abaecin* or *apidaecin 1* in both seasons. Change in *defensin 1* gene expression was observed after infection with gram-negative bacteria *E*. *coli* also by Richard et al. [43], who suggested possible cross-talk among signaling pathways regulating the expression of AMPs. In the honey bees injected with heat-killed *S*. *marcescens*, we report a similar pattern in Defensin 1 concentration with a slower rise in both summer and winter populations, with peaks at 22.9 and 25.8 hours, respectively.

Abaecin and Hymenoptaecin are effective against both, gram-positive and gram-negative bacteria [35, 36]. It was also reported that they are regulated by the Imd pathway [44], which is activated mostly by membrane components of gram-negative bacteria. In our study, *hymenoptaecin* shows the highest gene expression of the four studied peptides, which is in agreement with our previous study describing immune reaction after 24 hours post-treatment [3]. In addition, the high relative gene expression is followed by a high relative concentration in the hemolymph, which exceeds levels of Abaecin and Defensin 1, corresponding with the results of previous studies [24, 45].

Previously, a higher hemocyte concentration in the winter honey bees compared to the summer bees was reported [2]. This study confirmed the previous observation using flow cytometry and hemolymph samples of 10-day-old honey bees. Despite the higher concentration of hemocytes, winter bees seem to rely primarily on humoral immunity. Gätschenberger et al. [33] observed melanized nodules created only in the hemolymph of the summer and not the winter honey bees injected with live *E*. *coli*. Our model also shows shifts in hemocyte concentration only in the summer bee population. Azzami et al. [45] reported that the first nodules appeared in summer honey bees at 2 hours after injection of live *E*. *coli*, and the maximal count of nodules was reached at 6 hours post-treatment. In this study, the concentration of hemocytes in summer bees was the highest at 5.9 hours after injection with heat-killed *S*. *marcescens* and decreased over time. It could be suggested that more hemocytes are released into the hemolymph within a few hours after bacterial challenge, followed by their depletion in the immune reaction, as observed in various insect species [46, 47]. We also suggest that summer bees utilize both cellular and humoral immunity, while winter honeybees rely predominantly on humoral immunity. More research is needed, as there is no information about the changes in hemocyte populations in summer bees challenged with bacteria. In addition, the functional tests analyzing phagocytosis and nodulation would shed more light on the fate of these hemocytes.

In conclusion, Bayesian statistics provide a powerful tool for integrating prior knowledge, experimental data, and uncertainty estimation, making them particularly well-suited for understanding the dynamics of the immune system and its components. This approach can update predictions continuously as new data becomes available, enhancing the accuracy and reliability of the results. In this study, humoral defense both in summer and winter bee populations was observed; however, the fundamental parameter of cellular immunity, the hemocyte concentration, responded to heat-killed bacteria only in summer bees. It could be suggested that cellular immune defense is active mainly in summer bees, while winter bees rely mostly on humoral immune mechanisms. Moreover, our results established the eligible time points for studies focused on the humoral immunity of honey bees. These findings can be applied when using heat-killed bacteria as immune stimulators in future studies focused on the induction of honey bee immunity.

## Supporting information

S1 File ELISA buffers and reagents. (DOCX)

S2 File Python script. (ZIP)

S3 File Original data. (ZIP)

S1 Table Primer sequences and the corresponding target gene names used for PCR reactions. (DOCX)

S2 Table Values of the ampl and loc with 2.5–97.5% dispersion, i.e. 95% HDI. (XLSX)

S1 Fig Bayesian model of antimicrobial activity dynamics measured as lysozyme equivalent (mg/ml). Comparison of four experimental groups: Control (CTRL; green), CO2 (CO2; yellow), PBS (PBS; red), and Bacteria (BACTERIA; purple). (A) Summer and (B) winter honey bee population.
(TIF)

S2 Fig Bayesian model showing dynamics of abaecin relative gene expression and its relative peptide concentration in the hemolymph. Comparison of four experimental groups: Control (CTRL; green), CO2 (CO2; yellow), PBS (PBS; red), and Bacteria (BACTERIA; purple). Abaecin relative gene expression of (A) summer and (B) winter honey bee population. Abaecin relative peptide concentration of (C) summer and (D) winter honey bee population.
(TIF)

S3 Fig Bayesian model showing dynamics of apidaecin 1 relative gene expression and its peptide concentration in the hemolymph. Comparison of four experimental groups: Control (CTRL; green), CO2 (CO2; yellow), PBS (PBS; red), and Bacteria (BACTERIA; purple). Apidaecin 1 relative gene expression of (A) summer and (B) winter honey bee population. Apidaecin 1 peptide concentration of (C) summer and (D) winter honey bee population.
(TIF)

S4 Fig Bayesian model showing dynamics of defensin 1 relative gene expression and its relative peptide concentration in the hemolymph. Comparison of four experimental groups: Control (CTRL; green), CO2 (CO2; yellow), PBS (PBS; red), and Bacteria (BACTERIA; purple). Defensin 1 relative gene expression of (A) summer and (B) winter honey bee population. Defensin 1 relative peptide concentration of (C) summer and (D) winter honey bee population.
(TIF)

S5 Fig Bayesian model showing dynamics of hymenoptaecin relative gene expression and its relative peptide concentration in the hemolymph. Comparison of four experimental groups: Control (CTRL; green), CO2 (CO2; yellow), PBS (PBS; red), and Bacteria (BACTERIA; purple). Hymenoptaecin relative gene expression of (A) summer and (B) winter honey bee population. Hymenoptaecin relative peptide concentration of (C) summer and (D) winter honey bee population.
(TIF)

S6 Fig Bayesian model of hemocyte concentration (circulating hemocytes/μl of hemolymph) dynamics measured by flow cytometry. Comparison of four experimental groups: Control (CTRL; green), CO2 (CO2; yellow), PBS (PBS; red), and Bacteria (BACTERIA; purple). (A) Summer and (B) winter honey bee population.
(TIF)
