# Supplementary material for: Modeling seasonal immune dynamics of honey bee (Apis mellifera L.) response to injection of heat-killed Serratia marcescens
Source: PLoS One. 2024 Oct 4;19(10):e0311415. doi: 10.1371/journal.pone.0311415 (PMC11452037; doi:10.1371/journal.pone.0311415)
Supplement: S1 File — (DOCX) [file pone.0311415.s001.docx]

**Coating buffer**

1.272 g Na_2_CO_3_ and 1.512 g NaHCO_3_ was dissolved in 300 ml of deionized H_2_O, pH adjusted by 1M HCl to 9.6 and stored at 4 °C.

**10× washing buffer**

43.82 g NaCl, 2.08 g NaH_2_PO_4_·H_2_O and 0.74 g Na_2_HPO_4_ was dissolved in 500 ml of deionized H_2_O, pH adjusted with 1M NaOH to 7.5, autoclaved and stored at 4 °C.

**1× washing buffer + Tween 20 (ready to use)**

50 ml of 10× washing buffer was diluted with 450 ml of deionized H_2_O and 500 µl of Tween 20 was added to the solution.

**ELISA substrate reagents**

- 0.2 M Na_2_HPO_4_: 1.78 g of Na_2_HPO_4_·2H_2_O was dissolved in 50 ml of deionized H_2_O and stored at 4°C.
- 0.1M citric acid: 2.1 g of citric acid monohydrate was dissolved in smaller volume of deionized water and then brought up to the total volume of 100 ml and stored at 4°C.
- Phospho-citrate with sodium perborate buffer: 24 ml of 0.2 M Na_2_HPO_4_ was mixed with 26 ml of 0.1 M citric acid, pH adjusted to 4.6 by NaOH. Then 0.02 g of sodium perborate was added and stored at 4 °C in a polyethylene flask.
- 100 mM 3,3’,5,5’–tetramethylbenzidine (TMB): 24 mg of TMB was dissolved in 1 ml of DMSO.

**ELISA substrate (ready-to-use solution)**

One ml of phospho-citrate with sodium perborate buffer was mixed with 25 µl of 100 mM TMB (dilution 1:40) and stored in the dark. The substrate was always prepared fresh approx. 1 hour before use.
