# Supplementary material for: Modeling seasonal immune dynamics of honey bee (Apis mellifera L.) response to injection of heat-killed Serratia marcescens
Source: PLoS One. 2024 Oct 4;19(10):e0311415. doi: 10.1371/journal.pone.0311415 (PMC11452037; doi:10.1371/journal.pone.0311415)
Supplement: S2 File — (ZIP) [file pone.0311415.s002.zip › Script/analysis.pdf]

analysis

February 2, 2024

[ ]:
```
import numpy as np
import pandas as pd
import pymc as pm
import matplotlib.pyplot as plt
import seaborn as sns
import arviz as az
import glob
```

[ ]:
```
plt.style.use('seaborn-v0_8-whitegrid')
plt.rcParams['figure.figsize'] = [15, 5]
sns.set_context("paper", font_scale=1.5, rc={"font.size": 15, "axes.titlesize":␣
↪15, "axes.labelsize": 17})
```

[ ]:
```
N_POPS = 2
N_GROUPS = 4

POP_FROM_CODE = {0: 'Summer', 1: 'Winter'}
GROUP_FROM_CODE = {0: 'BACTERIA', 1: 'CO2', 2: 'CTRL', 3: 'PBS'}

POP_TO_CODE = {POP_FROM_CODE[k]: k for k in POP_FROM_CODE}
GROUP_TO_CODE = {GROUP_FROM_CODE[k]: k for k in GROUP_FROM_CODE}

POP_ORDER = ['Winter', 'Summer']
GROUP_ORDER = ['CTRL', 'CO2', 'PBS', 'BACTERIA']

POP_COLORS = {'Winter': '#1F77B4', 'Summer': '#FF7F0F'}
GROUP_COLORS = {'CTRL': '#b2df8a', 'CO2': '#ffd92f', 'PBS': '#b15928',␣
↪'BACTERIA': '#6a3d9a'}

assert N_POPS == len(POP_FROM_CODE)
assert N_GROUPS == len(GROUP_FROM_CODE)
# TODO...
```

$$f(x) = \frac{1}{1 + exp(-x)}$$

$$y(x;\text{ampl},\text{loc},\text{scale}) = 4 \cdot \text{ampl} \cdot f\left(\frac{x-\text{loc}}{\text{scale}}\right) \cdot \left(1 - f\left(\frac{\dots}{\dots}\right)\right)$$

[ ]:
```python
logistic = lambda x: 1 / (1 + np.exp(-x))

def hill(t, ampl, loc, scale):
    f = logistic((t-loc) / scale)
    return ampl * 4 * f * (1-f)
```

[ ]:
```python
t = np.linspace(0, 30, 500)

for s, ls in zip([0.1, 1, 3], ["-", "-.", "--"]):
    plt.plot(
        t,
        hill(t, ampl=5, loc=10, scale=s),
        label=f"ampl=5, loc=10, scale={s:.1f}",
        lw=2,
        ls=ls
    )

plt.xticks(np.arange(0, 30, 2))
plt.yticks(np.arange(0, 6+1, 1))
plt.grid(alpha=0.5)
plt.xlim([0, 30])
plt.ylim([0, 6])
plt.title("Function being fitted")
plt.xlabel("Time (hours)")
plt.ylabel("Measurement")
plt.legend()
plt.savefig("Fig1.png", dpi=300)
plt.show()
```

[ ]:
```python
file_paths = glob.glob('./data-unified-format/*.csv')
file_paths
```

[ ]:
```python
EXPECTED_COLUMNS = ['Population', 'Group', 'Hours pi', 'Measurement']

def load_data(pth: str) -> pd.DataFrame:
    """Expects all of `EXPECTED_COLUMNS`. Last column is interpreted as a␣
↪signal."""
    measurement_name = pth.split('/')[-1].split('.')[0]

    df = pd.read_csv(pth, sep=';')
    df = df[EXPECTED_COLUMNS]
    # df = df.loc[:, ~df.columns.str.contains('^Unnamed')]
```

```
    # Vsechny skupiny prevedu na uppercase.
    df['Group'] = df['Group'].str.upper()
    df['Group'] = df['Group'].str.replace('BAKTERIE', 'BACTERIA')

    if df['Measurement'].dtype.kind == 'O':
        # Asi obsahuje desetinnou carku misto tecky.
        # Nahradim carku teckou a pretypuji na float.
        df['Measurement'] = df['Measurement'].str.replace(',', '.').
↪astype(float)

    df = df.dropna(how='any')

    return df, measurement_name
```

[ ]:
```
data = dict()

for pth in file_paths:
    df, signal_column_name = load_data(pth)
    data[signal_column_name] = df

data["230922-hemocytes_revised"]["Measurement"] /= 1000
```

[ ]:
```
for s in data:
    print(s)
```

[ ]:
```
REACTIONS_FOR_ANALYSIS = [
    "210811-ELISA-Hymenoptaecin_absorbance",
    "210702-Antimicrobial_activity",
    "210205-Hymenoptaecin",
    "210811-ELISA-Defensin1_absorbance",
    "210603-Apidaecin-concentration_in_hemolymph",
    "210205-Abaecin",
    "210205-Def2",
    "210205-Def1",
    "210205-Apidaecin",
    "210811-ELISA-Abaecin_absorbance",
    "230922-hemocytes_revised"
]
```

[ ]:
```
def create_prob_model(df: pd.DataFrame):

    df['Group-codes'] = [GROUP_TO_CODE[k] for k in df['Group']]
    df['Pop-codes'] = [POP_TO_CODE[k] for k in df['Population']]

    coords = {
        "population": POP_FROM_CODE.values(),
        "group": GROUP_FROM_CODE.values(),
```

```
        "measurement": df.index,
    }

    with pm.Model(coords=coords) as model:
        ampl = pm.Lognormal('ampl', mu=np.log(df['Measurement'].mean()),␣
↪sigma=2, dims=["group", "population"])
        loc = pm.Normal('loc', mu=18, sigma=5, dims=["group", "population"])
        scale = pm.HalfNormal('scale', sigma=10, dims=["group", "population"])
        sd = pm.HalfNormal('sd', sigma=10, dims=["group", "population"])

        mu = hill(
            t=df['Hours pi'].values,
            ampl=ampl[df['Group-codes'].values, df['Pop-codes'].values],
            loc=loc[df['Group-codes'].values, df['Pop-codes'].values],
            scale=scale[df['Group-codes'].values, df['Pop-codes'].values]
        )

        obs = pm.Normal(
            'observations',
            mu=mu,
            sigma=sd[df['Group-codes'].values, df['Pop-codes'].values],
            observed=df['Measurement'].values,
            dims="measurement"
        )

    return model
```

[ ]:
```
models = dict()

for s in REACTIONS_FOR_ANALYSIS:
    models[s] = create_prob_model(data[s])
```

[ ]:
```
pm.model_to_graphviz(models['210702-Antimicrobial_activity'])
```

[ ]:
```
sampling_data = dict()

# for s in [REACTIONS_FOR_ANALYSIS[0], REACTIONS_FOR_ANALYSIS[-1]]:
for s in REACTIONS_FOR_ANALYSIS:
    with models[s]:
        sampling_data[s] = pm.sample(1000, tune=500)
```

[ ]:
```
sampling_data[s].posterior
```

[ ]:
```
Q = 0.975

out = []
```

```
for s in sampling_data:
    idata = sampling_data[s]
    for pop in POP_ORDER:
        for group in GROUP_ORDER:
            idata_selection = idata.sel(group=group, population=pop)
            loc_posterior = az.extract(idata_selection, group='posterior',␣
 ↪var_names='loc')
            ampl_posterior = az.extract(idata_selection, group='posterior',␣
 ↪var_names='ampl')

            row = {
                'marker': s,
                'population': pop,
                'group': group,
                f'loc-{(1-Q)*100:.1f}%': loc_posterior.quantile(q=1-Q).values,
                'loc-mean': loc_posterior.mean().values,
                f'loc-{Q*100:.1f}%': loc_posterior.quantile(q=Q).values,
                f'ampl-{(1-Q)*100:.1f}%': ampl_posterior.quantile(q=1-Q).values,
                'ampl-mean': ampl_posterior.mean().values,
                f'ampl-{Q*100:.1f}%': ampl_posterior.quantile(q=Q).values,
            }
            out.append(row)

df_out = pd.DataFrame(out)

FLOAT_COLS = ["loc-2.5%", "loc-mean", "loc-97.5%", "ampl-2.5%", "ampl-mean",␣
 ↪"ampl-97.5%"]

df_out[FLOAT_COLS] = df_out[FLOAT_COLS].astype(float)
df_out.round(2).to_excel('output.xlsx')

df_out.head(8)
```

[ ]:

```
Q = 0.95

for title in sampling_data:
    idata = az.extract(sampling_data[title])

    fig, ax = plt.subplots(1, 2, sharey=True, figsize=[15, 5])

    kwargs = dict(
        data=[idata.sel(population=pop) for pop in POP_ORDER],
        model_names=POP_ORDER,
        colors=[POP_COLORS[pop] for pop in POP_ORDER],
        coords=dict(group=GROUP_ORDER),
        hdi_prob=Q,
        combine_dims={"draw", "chain", "sample"},
```

```
        combined=True,
    )

    az.plot_forest(var_names=["ampl"], ax=ax[0], **kwargs)
    az.plot_forest(var_names=["loc"], ax=ax[1], **kwargs)

    # Clean information about variable form yticklabels
    ax[0].set_yticklabels([text.get_text().split('[')[1].split(']')[0] for text␣
 ↪in ax[0].get_yticklabels()])

    ax[0].set_xlabel(f"ampl ({Q:.0%} HDI)")
    ax[0].set_title(None)
    # ax[0].set_xlim([0, 30])

    ax[1].set_xlabel(f"loc ({Q:.0%} HDI)")
    ax[1].set_title(None)

    # if not title.startswith("23"):
    ax[1].set_xlim([0, 35])

    fig.suptitle(title)
    fig.savefig(f'./export/forests/{title}.png', dpi=300, bbox_inches='tight')
    fig.savefig(f'./export/forests/{title}.eps', format='eps')
    fig.show(warn=False)
```

[ ]:
```
YLABELS = {
    "210702-Antimicrobial_activity": "Lysozyme equivalent (mg/ml)",
    "210205-Hymenoptaecin": "Relative gene expression to HKG",
    "210205-Abaecin": "Relative gene expression to HKG",
    "210205-Apidaecin": "Relative gene expression to HKG",
    "210205-Def1": "Relative gene expression to HKG",
    "210205-Def2": "Relative gene expression to HKG",
    "210811-ELISA-Hymenoptaecin_absorbance": "Absorbance (450 nm)",
    "210811-ELISA-Abaecin_absorbance": "Absorbance (450 nm)",
    "210603-Apidaecin-concentration_in_hemolymph": "Concentration (ng/µl)",
    "230922-hemocytes_revised": "Hemocytes/nl",
}

N_SPAGHETTI = 50

for s in sampling_data:
    idata = sampling_data[s]

    xx = np.linspace(data[s]['Hours pi'].min(), data[s]['Hours pi'].max(), 100)
    sample = az.extract(idata, num_samples=N_SPAGHETTI)

    for pop in POP_ORDER:
```

```
        for group in GROUP_ORDER:
            idata_select = az.extract(idata).sel(group=group, population=pop)
            sample_select = sample.sel(group=group, population=pop)
            am = sample_select['ampl']
            lo = sample_select['loc']
            sc = sample_select['scale']

            sns.scatterplot(
                data=data[s][data[s]['Population'] == pop],
                x='Hours pi',
                y='Measurement',
                hue='Group',
                palette=GROUP_COLORS,
                legend=False
            )

            plt.plot(
                xx, hill(xx, ampl=float(idata_select['ampl'].mean().values),
                        loc=float(idata_select['loc'].mean().values),
                        scale=float(idata_select['scale'].mean().values)),
                label=group, color=GROUP_COLORS[group], lw=3)
            for j in range(N_SPAGHETTI):
                plt.plot(
                    xx,
                    hill(xx, ampl=am[j].values, loc=lo[j].values, scale=sc[j].
 ↪values),
                    color=GROUP_COLORS[group],
                    alpha=0.1
                )

        plt.title(f'{s} - {pop}')
        plt.xlabel("Time (hours)")
        plt.ylabel(YLABELS.get(s, "Measurement"))

        plt.legend(loc="upper left")
        plt.savefig(f'./export/expression_in_time/{s}_{pop}.png', dpi=300)
        plt.savefig(f'./export/expression_in_time/{s}_{pop}.eps', format='eps')
        # plt.savefig(f'./export/expression_in_time/{s}_{pop}.svg')

        plt.show()
```

[ ]:

```

```

[ ]:

```

```
