# Supplementary material for: Modeling seasonal immune dynamics of honey bee (Apis mellifera L.) response to injection of heat-killed Serratia marcescens
Source: PLoS One. 2024 Oct 4;19(10):e0311415. doi: 10.1371/journal.pone.0311415 (PMC11452037; doi:10.1371/journal.pone.0311415)
Supplement: S1 Table — (DOCX) [file pone.0311415.s004.docx]

Table S1: Sequences of primers used for quantitative PCR analysis.

| Target gene | Size (bp) | 5´-3´ Sequence | | Tm | E | Reference |
| --- | --- | --- | --- | --- | --- | --- |
| *RPS-5* (40S ribosomal protein S5) | 115 | F | AATTATTTGGTCGCTGGAATTG | 54.7 | 1.842 | Evans, 2006 |
|  |  | R | TAACGTCCAGCAGAATGTGGTA | 58.4 |  |  |
| *EF-1α* | 153 | F | GGAGATGCTGCCATCGTTAT | 63.9 | 1.808 | Lourenço et al., 2008 |
|  |  | R | CAGCAGCGTCCTTGAAAGTT | 64.4 |  |  |
| *Apidaecin type 14* | 80 | F | TTTTGCCTTAGCAATTCTTGTTG | 60.0 | 1.794 | Simone et al., 2009 |
|  |  | R | GTAGGTCGAGTAGGCGGATCT | 63.4 |  |  |
| *Abaecin* | 72 | F | CAGCATTCGCATACGTACCA | 64.6 | 1.829 | Evans, 2006 |
|  |  | R | GACCAGGAAACGTTGGAAAC | 63.3 |  |  |
| *Defensin-*1 | 119 | F | TGCGCTGCTAACTGTCTCAG | 64.5 | 1.755 | Evans, 2006 |
|  |  | R | AATGGCACTTAACCGAAACG | 63.6 |  |  |
| *Hymenoptaeci*n | 200 | F | CTCTTCTGTGCCGTTGCATA | 64.0 | 1.807 | Evans, 2006 |
|  |  | R | GCGTCTCCTGTCATTCCATT | 64.0 |  |  |

The formula used for quantification of relative gene expression. The housekeeping gene corresponds to the reference gene, i.e. *RPS-5*, *EF-1 alpha*.


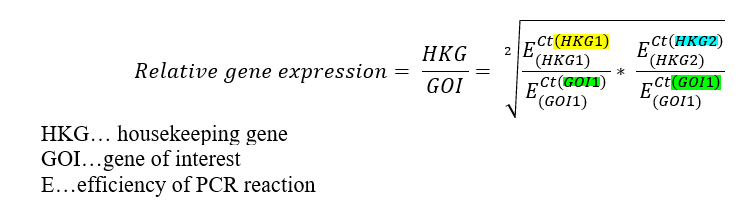


References

Evans, J., 2006. Beepth: an ordered quantitative-PCR array for exploring honey bee immunity and disease. J Invertebr Pathol 93, 135 - 139, DOI: [10.1016/j.jip.2006.04.004](https://doi.org/10.1016/j.jip.2006.04.004).

Lourenço, A., Pedro, Mackert, A., dos Santos Cristino, A., Simões, Z., Luz Paulino, 2008. Validation of reference genes for gene expression studies in the honey bee, Apis mellifera, by quantitative real-time RT-PCR. Apidologie 39, 372-385, DOI: 10.1051/apido:2008015.

Simone, M., Evans, J.D., Spivak, M., 2009. Resin collection and social immunity in honey bees. Evolution 63, 3016-3022, DOI: [10.1111/j.1558-5646.2009.00772.x](https://doi.org/10.1111/j.1558-5646.2009.00772.x).

Pfaffl, M.W., 2001. A new mathematical model for relative quantification in real-time RT–PCR. Nucleic Acids Research 29, , Page e45, DOI: [10.1093/nar/29.9.e45](https://doi.org/10.1093/nar/29.9.e45).
