# Supplementary figures and images for: Modeling seasonal immune dynamics of honey bee (Apis mellifera L.) response to injection of heat-killed Serratia marcescens
Source: PLoS One. 2024 Oct 4;19(10):e0311415. doi: 10.1371/journal.pone.0311415 (PMC11452037; doi:10.1371/journal.pone.0311415)

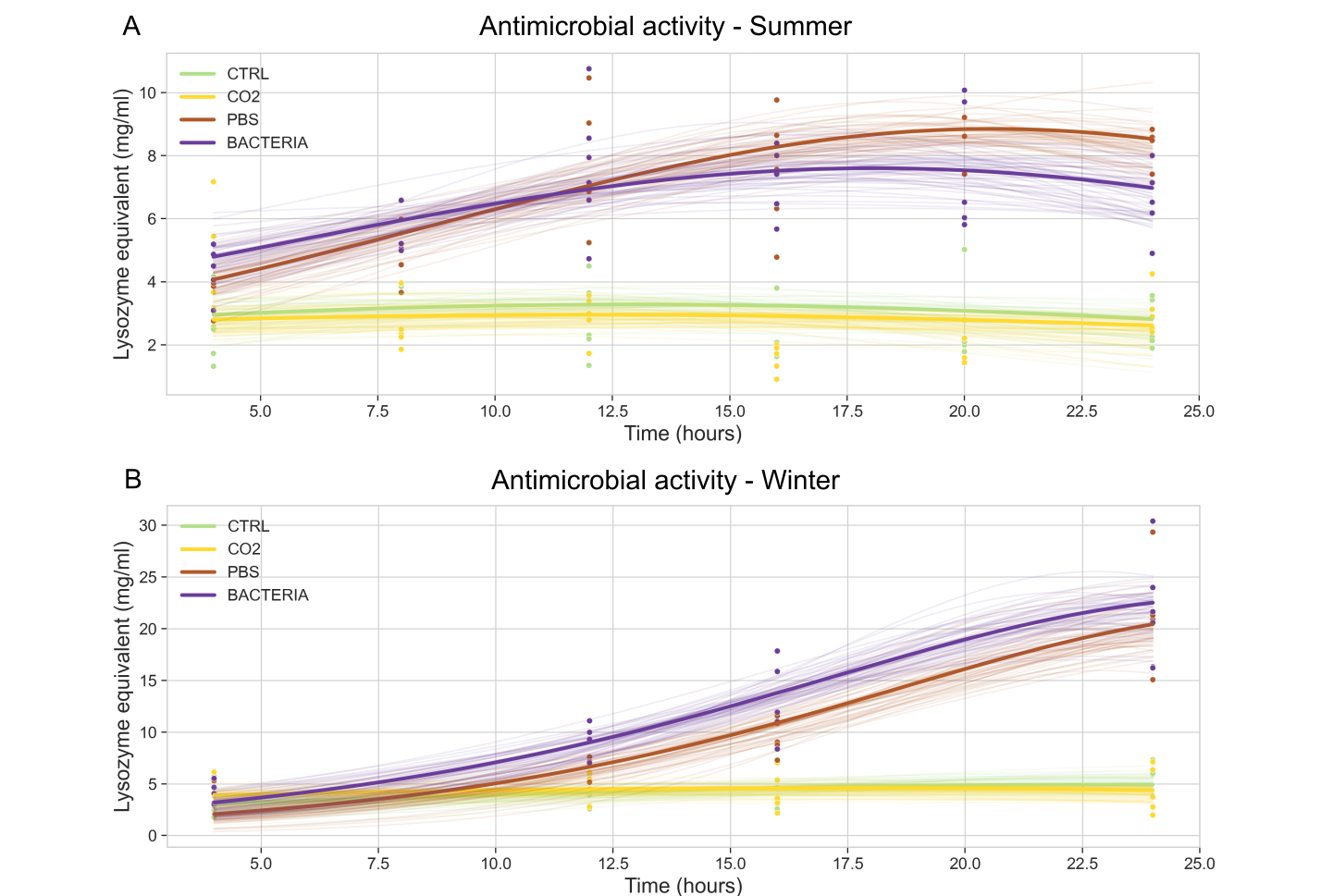

Supplement: S1 Fig — Comparison of four experimental groups: Control (CTRL; green), CO2 (CO2; yellow), PBS (PBS; red), and Bacteria (BACTERIA; purple). (A) Summer and (B) winter honey bee population. (TIF) [file pone.0311415.s006.tif]

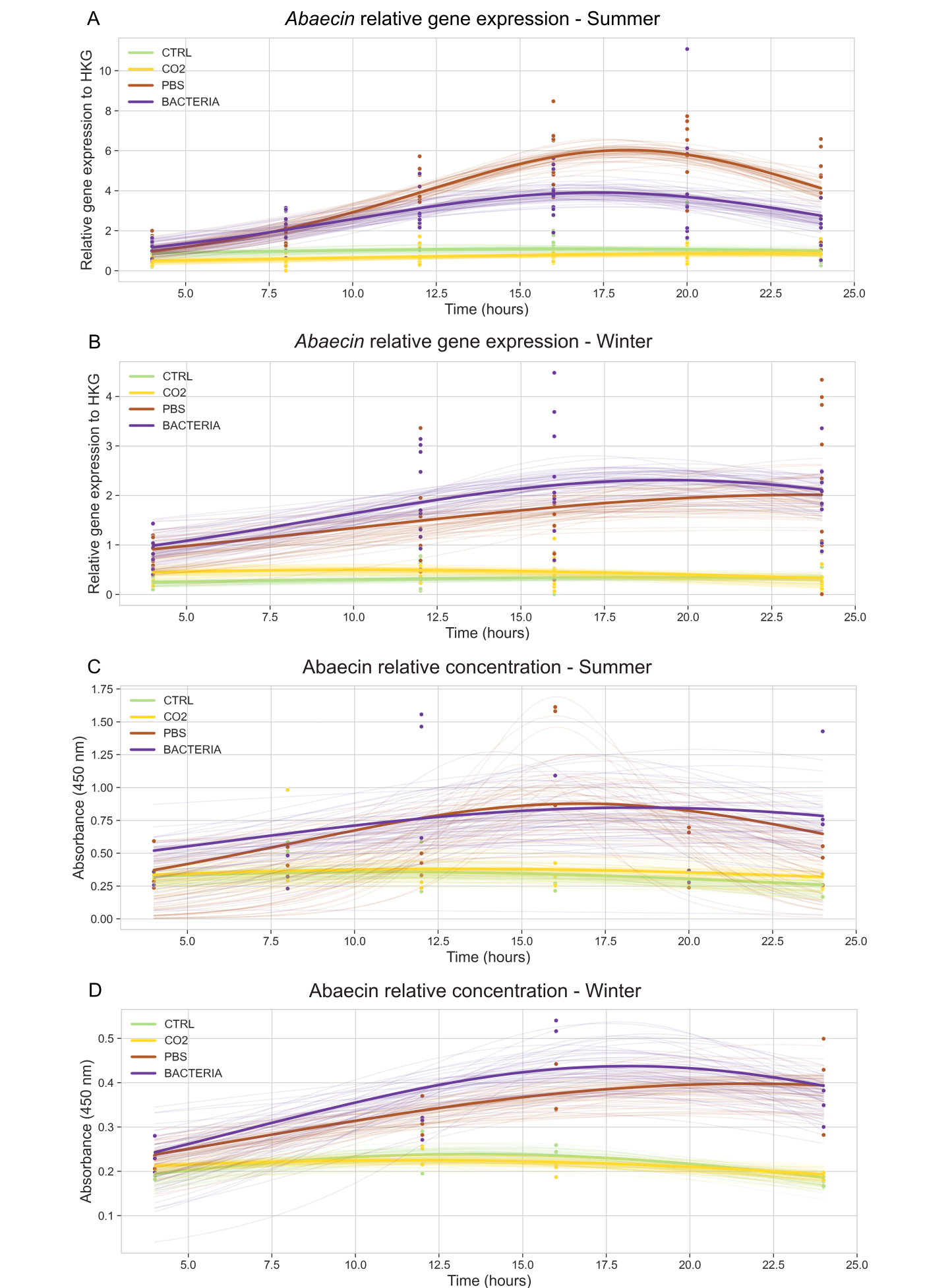

Supplement: S2 Fig — Comparison of four experimental groups: Control (CTRL; green), CO2 (CO2; yellow), PBS (PBS; red), and Bacteria (BACTERIA; purple). Abaecin relative gene expression of (A) summer and (B) winter honey bee population. Abaecin relative peptide concentration of (C) summer and (D) winter honey bee population. (TIF) [file pone.0311415.s007.tif]

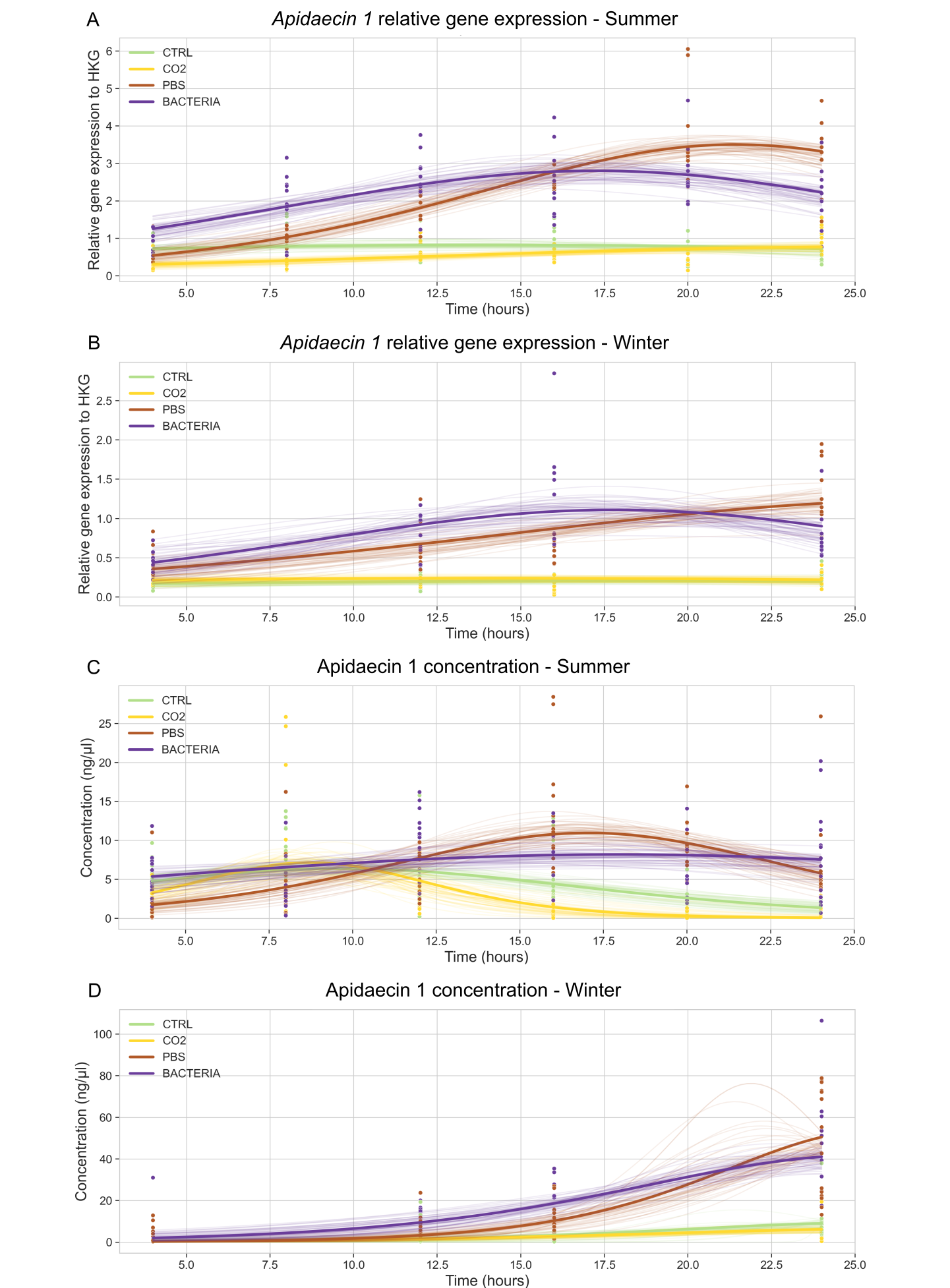

Supplement: S3 Fig — Comparison of four experimental groups: Control (CTRL; green), CO2 (CO2; yellow), PBS (PBS; red), and Bacteria (BACTERIA; purple). Apidaecin 1 relative gene expression of (A) summer and (B) winter honey bee population. Apidaecin 1 peptide concentration of (C) summer and (D) winter honey bee population. (TIF) [file pone.0311415.s008.tif]

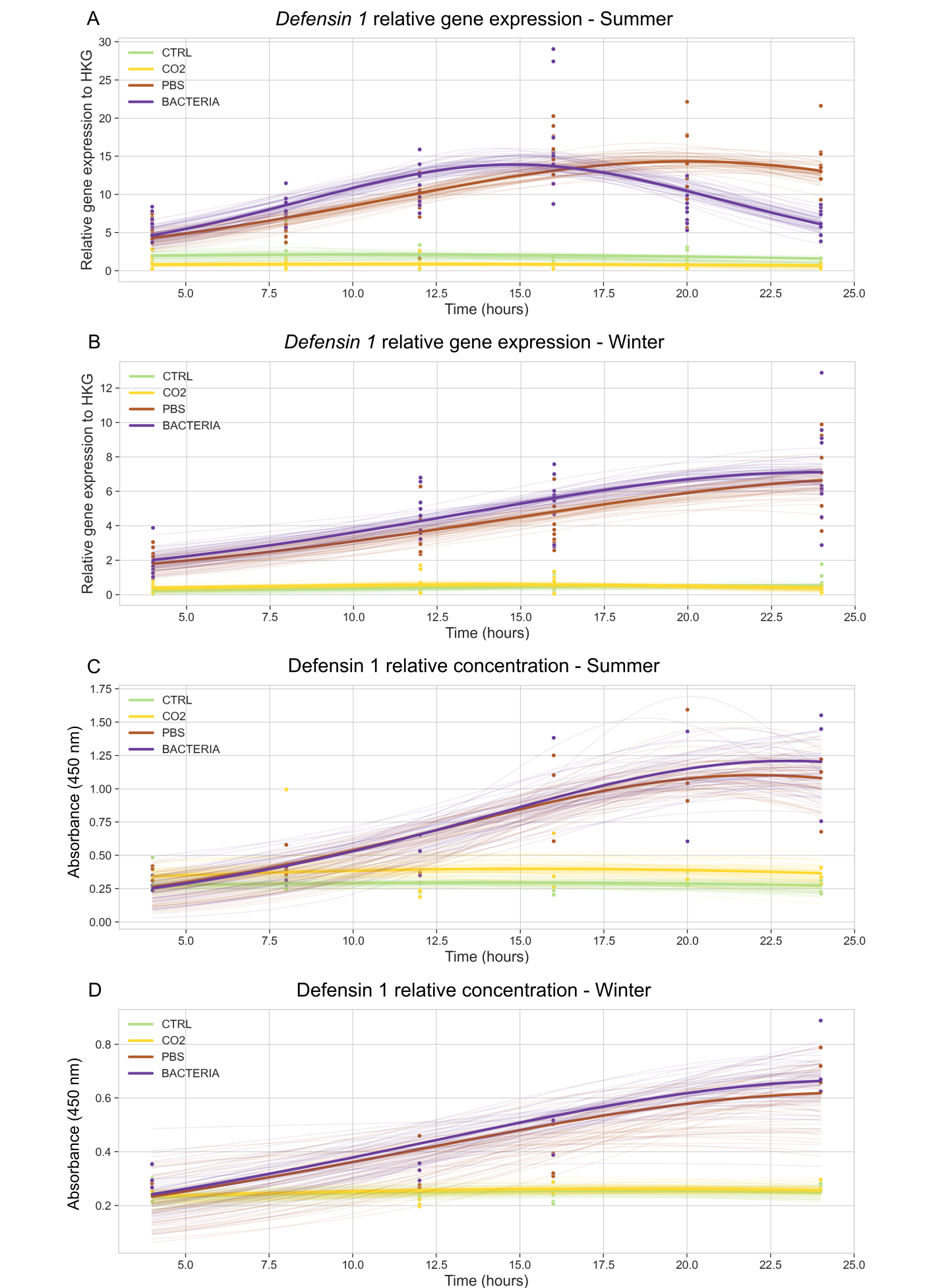

Supplement: S4 Fig — Comparison of four experimental groups: Control (CTRL; green), CO2 (CO2; yellow), PBS (PBS; red), and Bacteria (BACTERIA; purple). Defensin 1 relative gene expression of (A) summer and (B) winter honey bee population. Defensin 1 relative peptide concentration of (C) summer and (D) winter honey bee population. (TIF) [file pone.0311415.s009.tif]

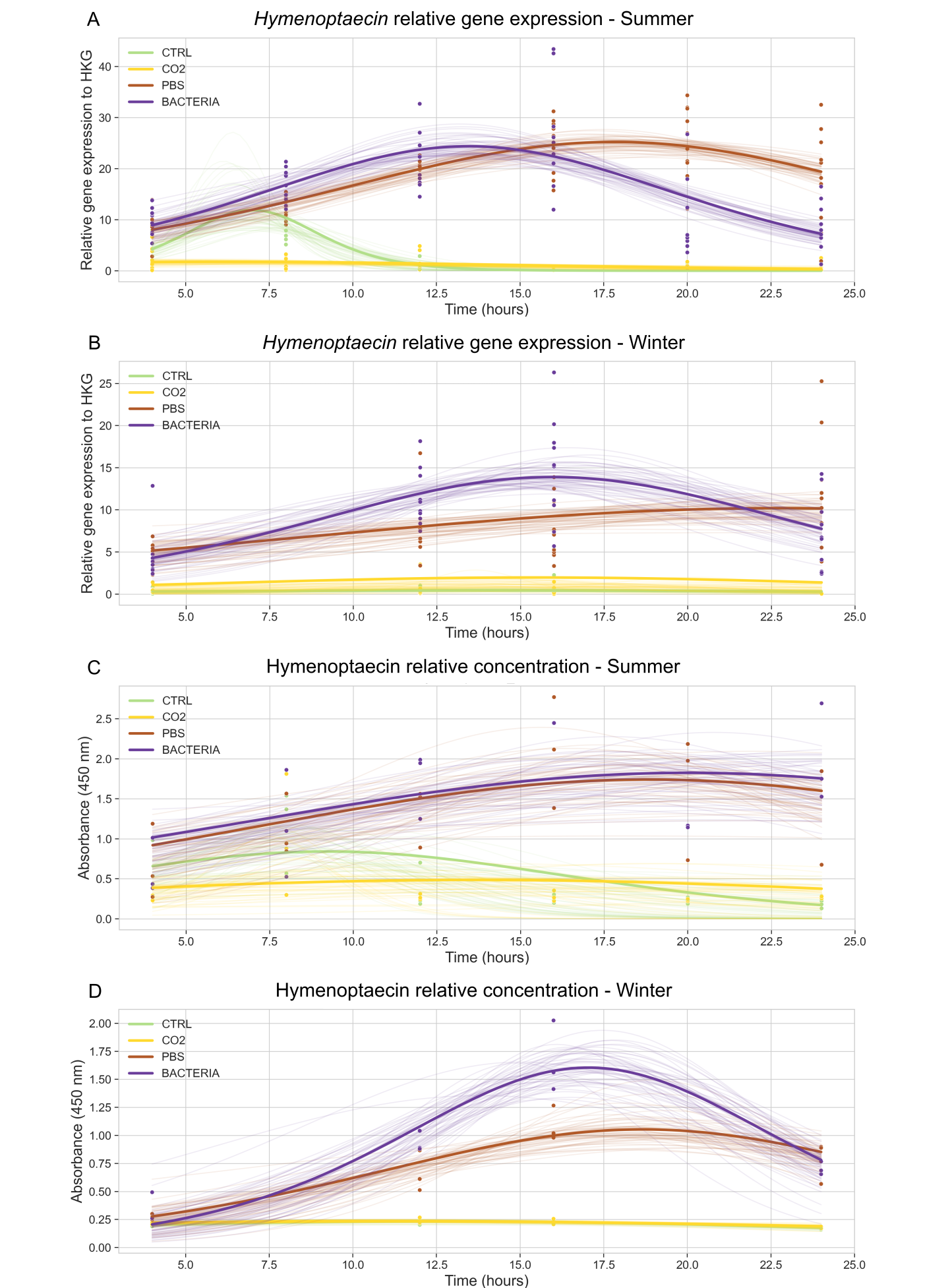

Supplement: S5 Fig — Comparison of four experimental groups: Control (CTRL; green), CO2 (CO2; yellow), PBS (PBS; red), and Bacteria (BACTERIA; purple). Hymenoptaecin relative gene expression of (A) summer and (B) winter honey bee population. Hymenoptaecin relative peptide concentration of (C) summer and (D) winter honey bee population. (TIF) [file pone.0311415.s010.tif]

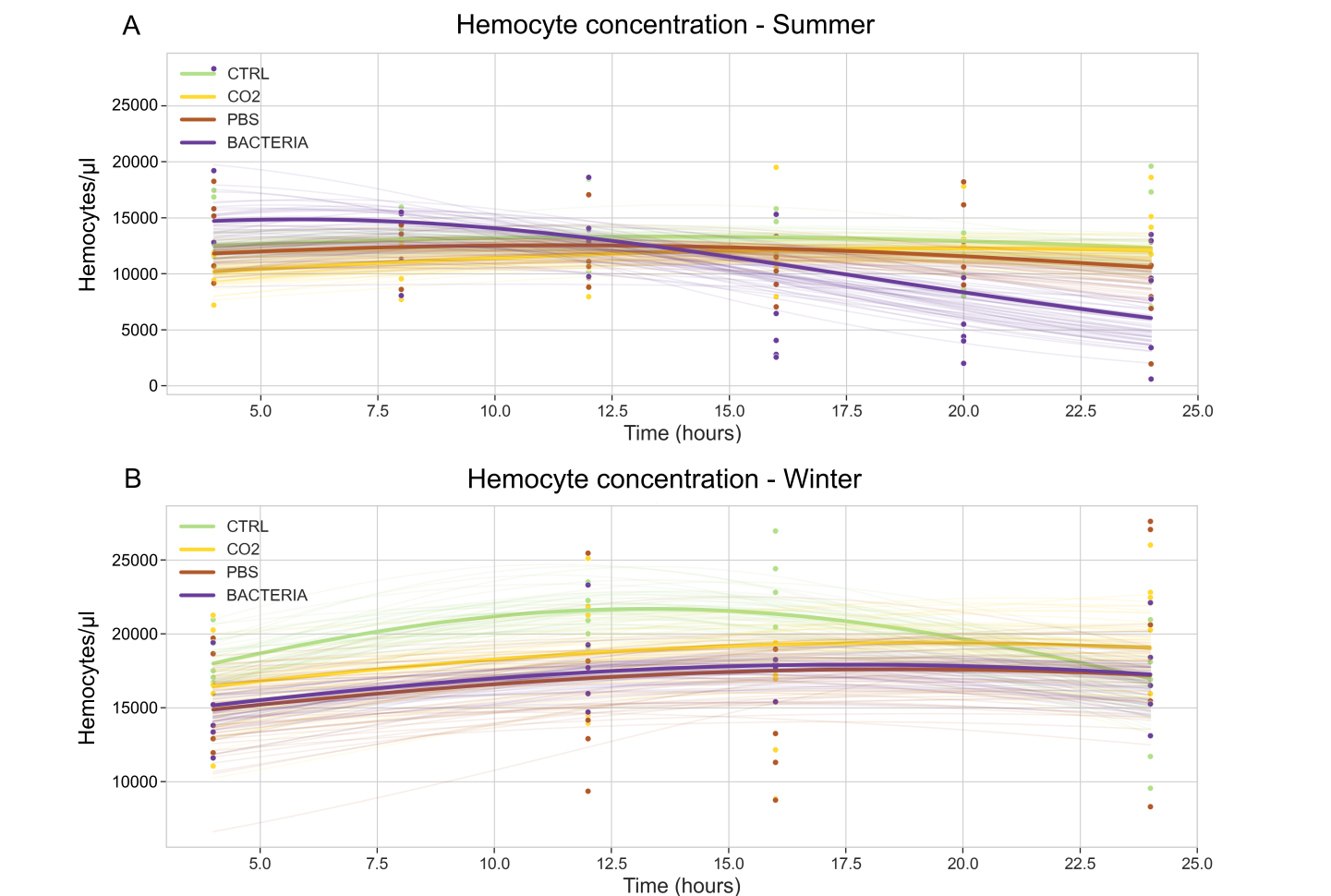

Supplement: S6 Fig — Comparison of four experimental groups: Control (CTRL; green), CO2 (CO2; yellow), PBS (PBS; red), and Bacteria (BACTERIA; purple). (A) Summer and (B) winter honey bee population. (TIF) [file pone.0311415.s011.tif]
